# Supplementary material for: Substrate recognition in Bacillus anthracis sortase B beyond its canonical pentapeptide binding motif and use in sortase-mediated ligation
Source: J Biol Chem. 2025 Mar 4;301(4):108382. doi: 10.1016/j.jbc.2025.108382 (PMC11987632; doi:10.1016/j.jbc.2025.108382)
Supplement: Supporting information [file mmc1.pdf]

Supporting Information for

Substrate recognition in *Bacillus anthracis* sortase B beyond its canonical pentapeptide binding motif and use in sortase-mediated ligation

Sophie N. Jackson, Jadon M. Blount, Kayla A. Croney, Darren E. Lee, Justin W. Ibershof, Caroline M. Ceravolo, Kate M. Brown, Noah J. Goodwin-Rice, Kyle M. Whitham, James McCarty, John M. Antos, Jeanine F. Amacher

Table of Contents:

Table S1. LC-MS analysis of N-terminal products from baSrtB nucleophile experiment. 2

Figure S1. Size exclusion chromatography, LmSrtB activity assay with alternative substrate sequence, and LC-MS analysis of SrtB assays. 3

Figure S2. Control activity assays for baSrtB. 4

Figure S3. X-ray diffraction of baSrtB crystals and AlphaFold structural models. 5

Figure S4. Comparison of baSrtB model with experimental saSrtB-NPQT* experimental structure (PDB 4LFD), and 6xHis-baSrtB AlphaFold3 model. 6

Figure S5. Control triplicate molecular dynamics simulations of SrtB bound to polyAla peptide. 7

Figure S6. Molecular dynamics simulations of SrtB AlphaFold2 models. 8

Experimental procedure for the synthesis of GGG-*biotin.* 9

Figure S7. Synthetic scheme and characterization of GGG-*biotin*. 10

Figure S8. LC-MS and SDS-PAGE characterization of mTurq-DNPKTGDEGGGG ligation to GGG-*biotin*. 11

Figure S9. LC-MS characterization of baSrtB enzyme-substrate adducts. 12

Sequences used for AlphaFold2 modeling. 13

References 14

Table S1. LC-MS analysis of N-terminal products from baSrtB nucleophile experiment.

Nucleophile	Product	Calculated (*m/z*)	Observed (*m/z*)
H_2O	Abz-DNPKT***-OH***	693.31	*not observed*
NH_2OH	Abz-DNPKT***-NHOH***	708.32	708.4
Gly-Gly-Gly	Abz-DNPKT**GGG**	864.38	864.5
Gly-NH_2	Abz-DNPKT**G-**NH_2	749.35	749.4
Ala-NH_2	Abz-DNPKT**A-**NH_2	763.37	763.5
D-Ala-NH_2	Abz-DNPKT**A-**NH_2	763.37	763.4
D-Ala	Abz-DNPKT**A**	764.35	764.4

Calculated and observed masses represent $[M+H]^+$ ions (monoisotopic). [-NH_2 = C-terminal primary amide].

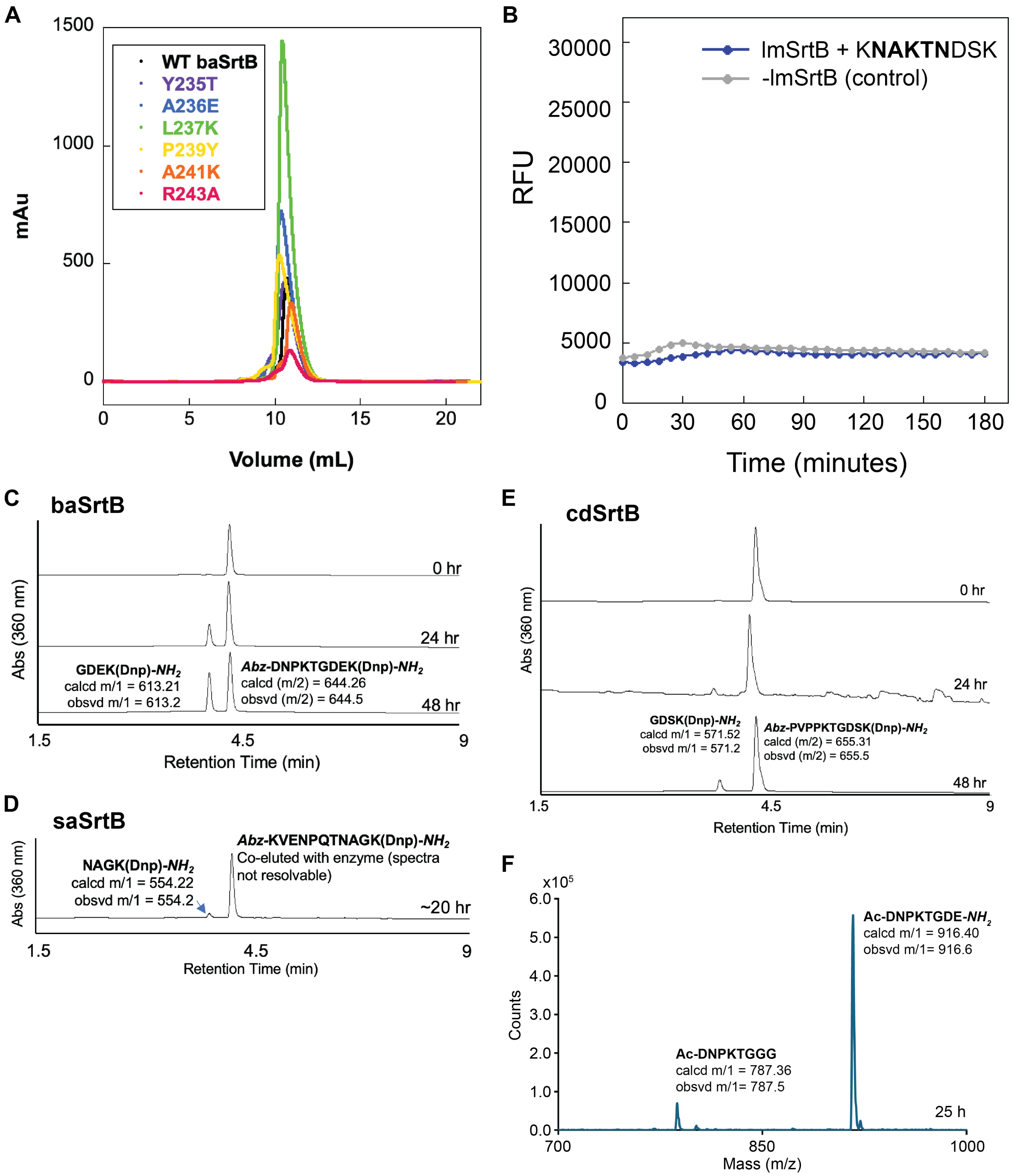

Figure S1. Size exclusion chromatography, LmSrtB activity assay with alternative substrate sequence, and LC-MS analysis of SrtB assays. (**A**) Analytical size exclusion chromatography elution profiles of selected baSrtB variants used in this study and wild-type. Differing concentrations of each enzyme were used to highlight the overlapping nature for all. This result is consistent with folded monomeric protein. (**B**) Averaged activity assays, in triplicate, of lmSrtB and KNAKTNDSK substrate sequence (from Lmo2185). The negative control (minus lmSrtB) is shown as well. (**C-E**) Analyses using liquid chromatography and mass spectrometry (LC-MS) of (**C**) baSrtB, (**D**) saSrtB, and (**E**) cdSrtB activity assays using peptide substrates. Peaks are labeled. (**F**) ESI-MS spectrum of a ligation reaction catalyzed by baSrtB, involving a peptide substrate lacking the Abz/Dnp labels and a triglycine (GGG) nucleophile (25 h at room temperature).

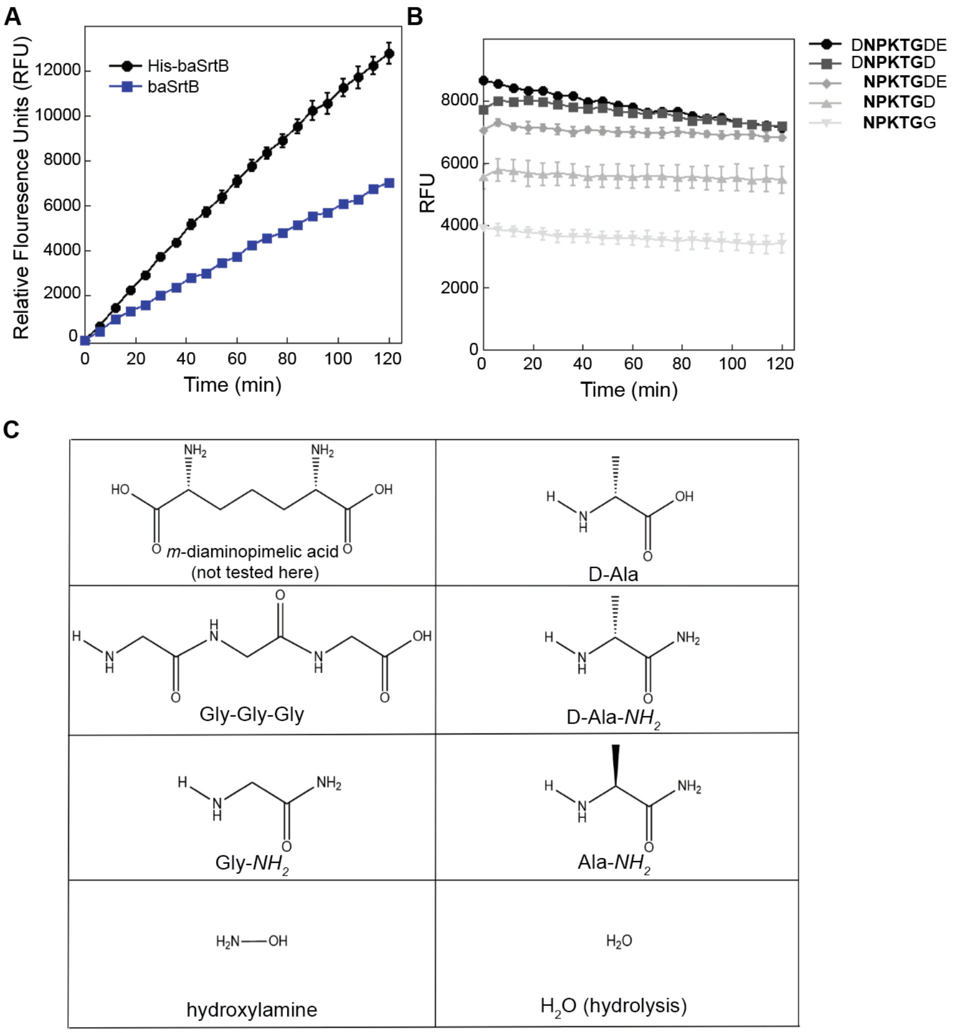

Figure S2. Control activity assays for baSrtB. (**A**) A peptide cleavage assay (using Abz-DNPKTGDE-K(Dnp) as the substrate) revealed that the 6xHis-baSrtB enzyme had approximately doubled activity at the t = 2 hr timepoint, as compared to the cleaved (-6xHis) baSrtB. These data represent triplicate technical replicates, with standard deviations shown. (B) Background fluorescence traces of the substrate peptides used. (**C**) The chemical structures of the potential baSrtB nucleophiles tested**.**

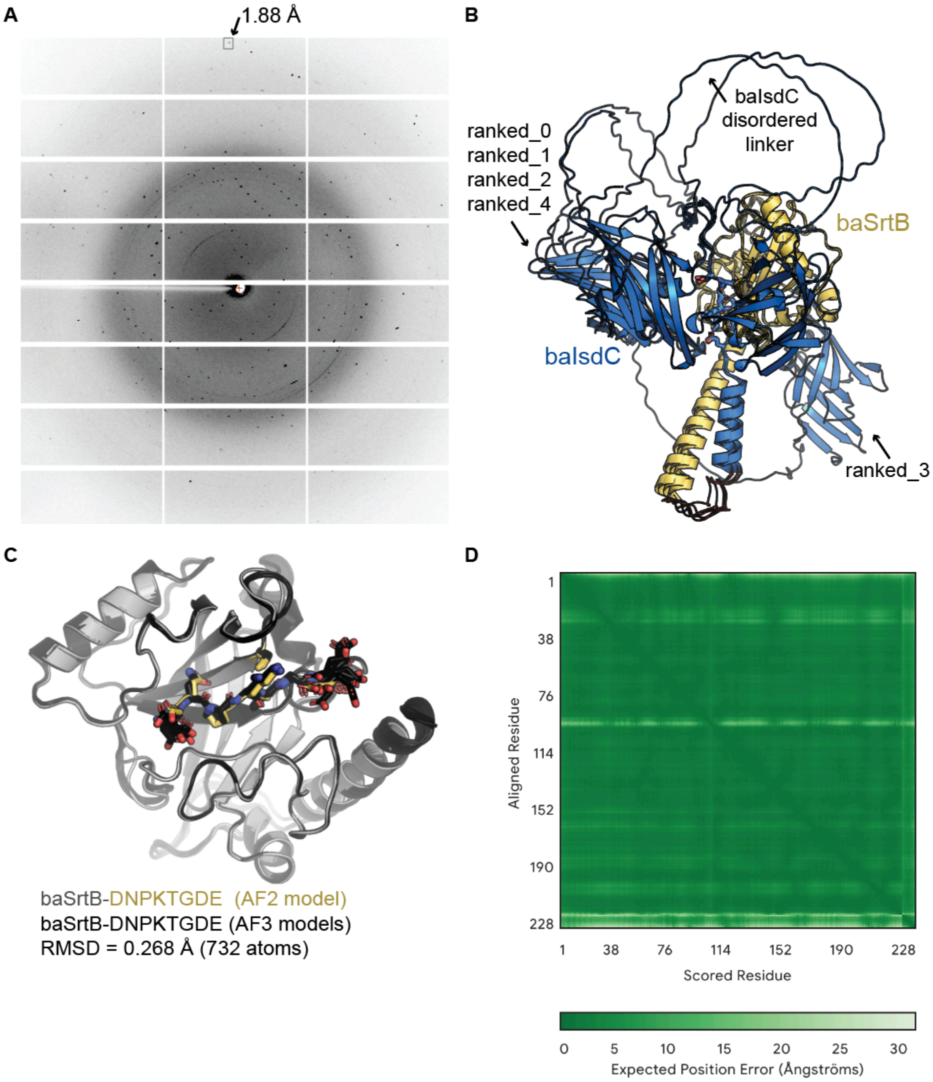

Figure S3. X-ray diffraction of baSrtB crystals and AlphaFold structural models. (**A**) X-ray diffraction pattern of baSrtB-DNPKTGDE crystals. Subsequent data processing and structure solution revealed that the peptide was not present in the crystals. (**B**) All 5 AlphaFold2 output models are shown for baIsdC-$(G_4S)_2$-baSrtB. Models are aligned using the full-length baSrtB sequence. An identified baIsdC disordered linker is labeled. (**C**) The five output models of baSrtB with the DNPKTGDE ligand, generated using AlphaFold3 (black cartoon models, with the catalytic Cys and peptide shown as sticks and colored by heteroatom, N=blue, O=red, S=gold, C=black), are very similar to the model generated using AlphaFold2 (gray cartoon, with the catalytic Cys and peptide shown as sticks and colored by heteroatom, here C=yellow). (**D**) The output PAE plot for the baSrtB-DNPKTGDE plot from the AlphaFold3 server revealed a high level of confidence for these models (ipTM = 0.77, pTM = 0.94).

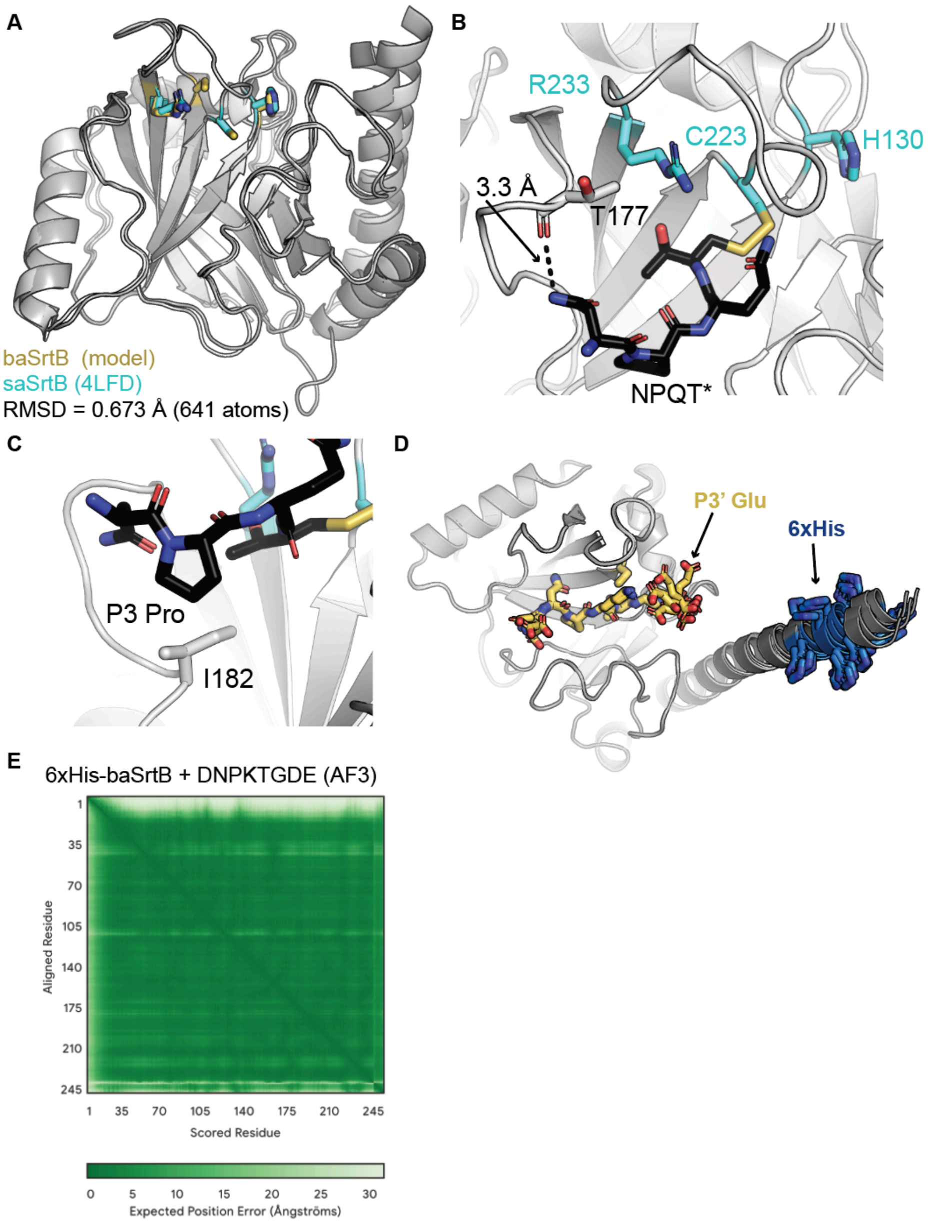

Figure S4. Comparison of baSrtB model with experimental saSrtB-NPQT* experimental structure (PDB 4LFD), and 6xHis-baSrtB AlphaFold3 model. For all, SrtB proteins are shown in gray cartoon representation, with the side chains of the catalytic triad shown as sticks and colored golden yellow (baSrtB) or cyan (saSrtB) and by atom (N=blue, S=yellow). The NPQT* peptidomimetic is shown as black sticks and colored by atom (O=red). The DNPKTGDE ligand is shown as yellow sticks and colored by heteroatom. (**A**) Alignment of the main chain atoms of the catalytic domains of baSrtB and saSrtB revealed an overall RMSD = 0.673 Å (641 atoms). (**B-C**) Specific interactions of NPQT* and saSrtB in the experimental structure are highlighted for the (**B**) P4 Asn and (**C**) P3 Pro residues. (**D**) The 6xHis-baSrtB with DNPKTGDE ligand model was generated using AlphaFold3. All 5 output models are shown, and the His-tag is highlighted (side chains shown as sticks and colored blue). Although not directly interacting in any of the models, the P3' Glu is in the vicinity of the 6xHis tag, indicated by black arrows and labeled. (**E**) The output PAE plot for the baSrtB-DNPKTGDE plot from the AlphaFold3 server revealed a high level of confidence for these models (ipTM = 0.76, pTM = 0.91).

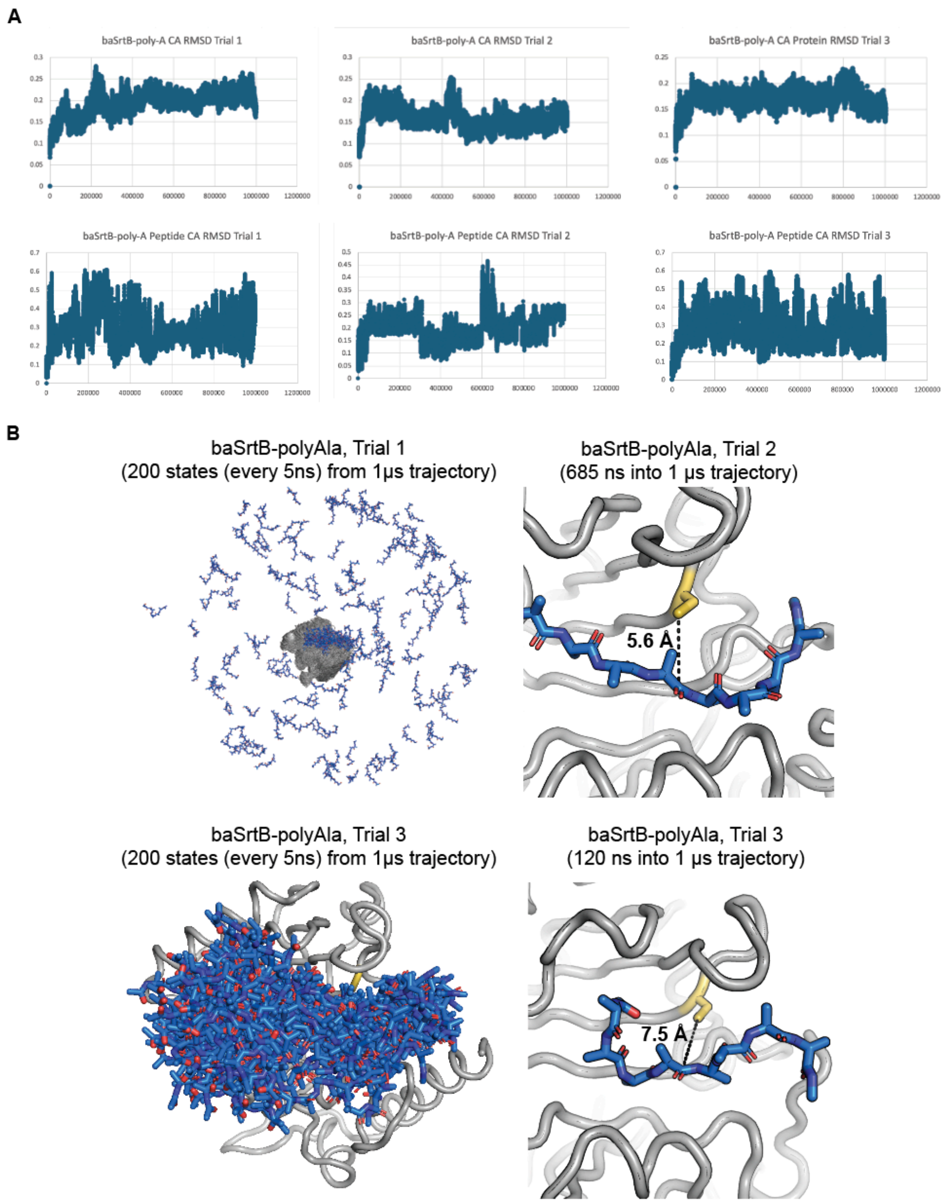

Figure S5. Control triplicate molecular dynamics simulations of SrtB bound to polyAla peptide. (**A**) Average RMSD values for the SrtB C_α atoms over the 1000 nsec trajectories are shown for each trajectory, as well as for the peptide ligand (sequence: AAAAAAAA). (**B**) Representative images for each of the triplicate MD simulations. For all, the baSrtB enzyme is shown in gray cartoon representation, with the catalytic Cys side chain as yellow sticks (C=yellow, S=gold). The polyAla peptide is shown as blue sticks (C=marine, N=blue, O=red). The peptide completely dissociated in Trial 1 (top left image) and shifted dramatically in the pocket in Trial 3 (bottom 2 images). The most stable of the replicate trajectories was Trial 2 (top right image), where the peptide largely stayed bound, although did move away from the catalytic Cys in the final 2/3rd of the simulation.

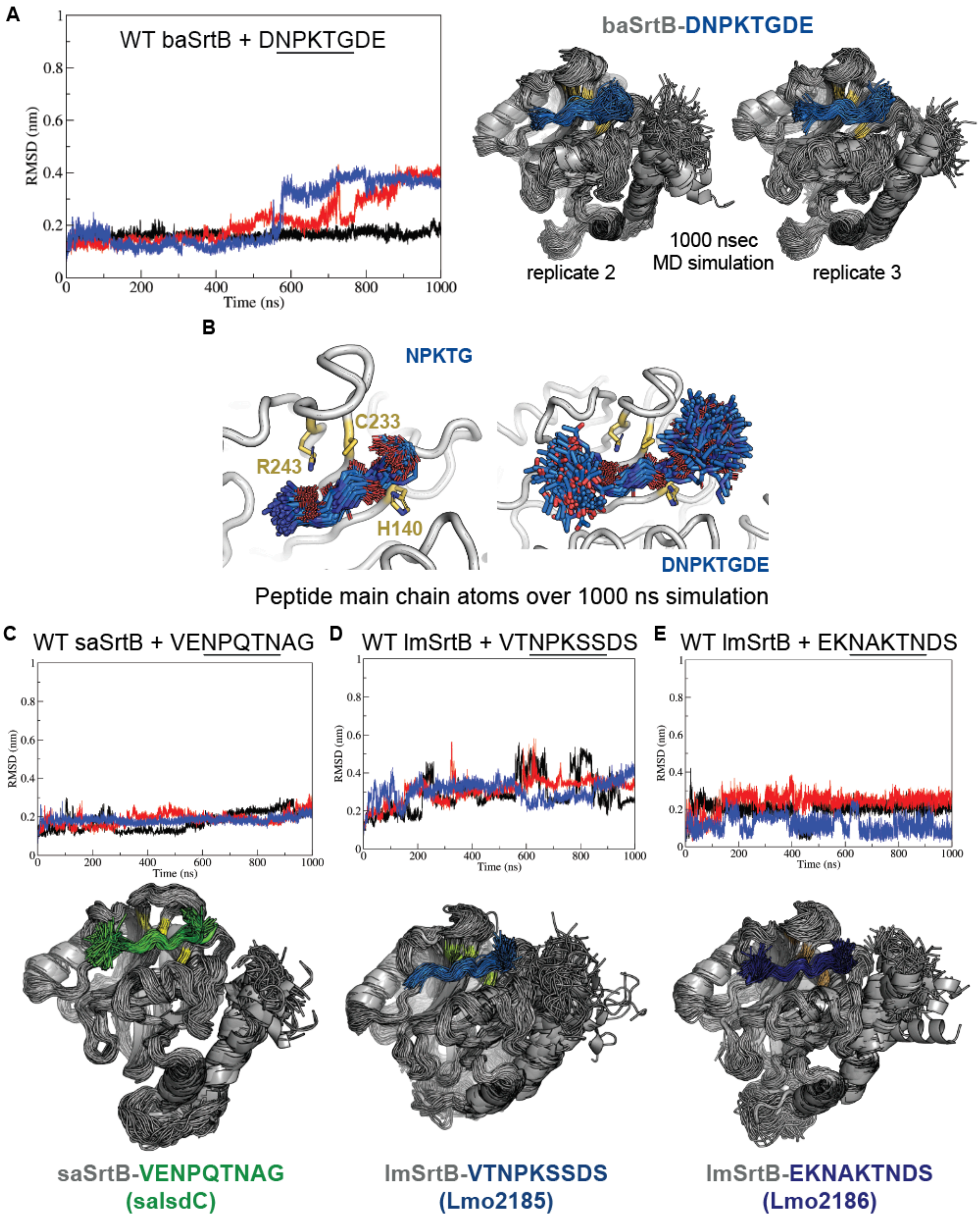

Figure S6. Molecular dynamics simulations of SrtB AlphaFold2 models. (**A, C-E**) Average RMSD values for the SrtB C$_\alpha$ atoms over the 1000 nsec trajectories are shown for (**A**) baSrtB, (**C**) saSrtB, (**D-E**) lmSrtB with different substrates. All 3 replicates are graphed. For baSrtB, 200 frames (every 5 nsec) of the remaining 2 replicates are aligned and shown as cartoons and colored as in **Figure 5A**. For saSrtB and lmSrtB, 200 frames (every 5 nsec) from only one replicate for each MD simulation is aligned and shown, with the peptide substrate colored as labeled. (**B**) The peptide backbone atoms are shown as sticks and colored by atom (N=blue, O=red). The peptides of 200 frames (every 5 sec) are aligned to highlight the stability of the pentapeptide motif, NPKTG (left), as compared to the P5 Asp, P2' Asp, and P3' Glu residues (DNPKTGDE, right). The side chain atoms of the catalytic triad residues are shown in golden yellow, colored by atom (S=yellow), and labeled.

Experimental procedure for the synthesis of GGG-*biotin*. The synthesis of GGG-*biotin* was achieved using a combination of manual solid phase peptide synthesis (SPPS), followed by a solution phase coupling of biotin (**Figure S7A**). Unless noted otherwise, all SPPS steps (washing, coupling, deprotection) were performed at room temperature and included gentle agitation on a bench-top rocking platform. Incorporation of the 2,4-dinitrophenyl (Dnp) chromophore to assist with estimation of peptide solution concentration was achieved using a commercially available lysine building block (Fmoc-Lys(Dnp)-OH) purchased from APExBIO. Biotin was purchased from Gold Biotechnology. All other materials, including standard Fmoc protected amino acids, Fmoc-Rink amide MBHA resin, and reagents for coupling, deprotection, and resin cleavage were obtained from commercial sources and used without further purification.

First, a 15 mL polypropylene synthesis vessel fitted with appropriate frits and inlet/outlet caps was loaded with 0.17 g (0.7 mmol) of Fmoc Rink amide MBHA resin (**S1**, 0.4 mmol/g). The resin was then washed/swollen with ~10 mL of N-methyl-2-pyrrolidinone (NMP) (3x, 10 min per wash). Next, the base-labile Fmoc group was removed with 10 mL of 20% piperidine in NMP (2x, 10 min per treatment), followed by washing with ~10 mL of NMP (3x, 5 min per wash). The resin was then elaborated through sequential coupling of Fmoc-Lys(Dnp)-OH, Fmoc-Lys(Boc)-OH, and Fmoc-Gly-OH. For each residue, a coupling solution consisting of Fmoc amino acid (0.3 mmol), O-(benzotriazol-1-yl)-N,N,N',N'-tetramethyluronium hexafluorophosphate (HBTU) (0.3 mmol), and N,N-diisopropylethylamine (DIPEA) (0.5 mmol) in ~5 mL of NMP was used. Following thorough mixing, the coupling solutions were added to the synthesis vessel containing the deprotected resin. Couplings were incubated for 1 h at room temperature. Following each coupling, the resin was washed with ~10 mL NMP (3x, 10 min per wash). The resin was then deprotected with ~10 mL of 20% piperidine in NMP (2x, 10 min per treatment), and washed with ~10 mL NMP (3x, 5 min per wash). Repeated cycles of coupling and deprotection were then used to assemble the target sequence, resulting in resin bound intermediate **S2** (**Figure S7A**). Following completion of the synthesis, the resin was washed with NMP (3x) and CH_2Cl_2 (3x). A 5 mL solution of 95:2.5:2.5 TFA/TIPS/H_2O was then used to cleave the peptide from the resin (2x, 30 min per treatment). The resin was not agitated during the cleavage step. The cleaved peptide (**S3**) solution was concentrated on a rotary evaporator, and the remaining residue was added dropwise to 35 mL of diethyl ether chilled over dry ice. The suspension was centrifuged at 4500 rpm for 5 minutes at 4 °C to collect precipitated peptide. The diethyl ether was decanted and the crude peptide was dried overnight. The identity of the crude peptide (**S3**) was confirmed by LC-ESI-MS ($[M+H]^+$ = 833.4 calcd, 833.4 obs). A portion of crude **S3** was then solubilized in NMP (100 mM stock concentration) and used without further purification.

For biotin installation, crude **S3** (8.3 mg, 10 µmol) was combined with 1.0 equivalent of biotin, 1.0 equivalent of HBTU, and 10 µL of DIPEA in NMP (310 µL total reaction volume). The reaction was incubated at room temperature for 30 min and then treated with piperidine (78 µL). The reaction was incubated for an additional 5 minutes to remove Fmoc, and then the GGG-biotin was purified directly from the reaction mixture by RP-HPLC. This was achieved using a Dionex Ultimate 3000 HPLC system equipped with a Phenomenex Luna 5 µM C18(2) 100 Å column (10 x 250 mm) [aqueous (95% water, 5% MeCN, 0.1% formic acid) / MeCN (0.1% formic acid) mobile phase at 4.0 mL/min, method: hold 10% MeCN 0.0-2.0 min, linear gradient of 10-90% MeCN 2.0-15.0 min, hold 90% MeCN 15.0-17.0 min, linear gradient of 90-10% MeCN 17.0-17.01 min, re-equilibrate at 10% MeCN 17.01-19.0 min)]. Pure fractions of GGG-biotin were pooled and lyophilized. Prior to use in sortase-mediated ligation, GGG-biotin was solubilized in DMSO and the solution concentration was estimated by diluting it into water (at least 100-fold) and using the absorbance of the Dnp chromophore at 365 nm (extinction coefficient = 17,300 $M^{-1}cm^{-1}$) (1, 2). The purity and identity of GGG-biotin in the stock solution was also confirmed by RP-HPLC and LC-MS (**Figure S7B**).

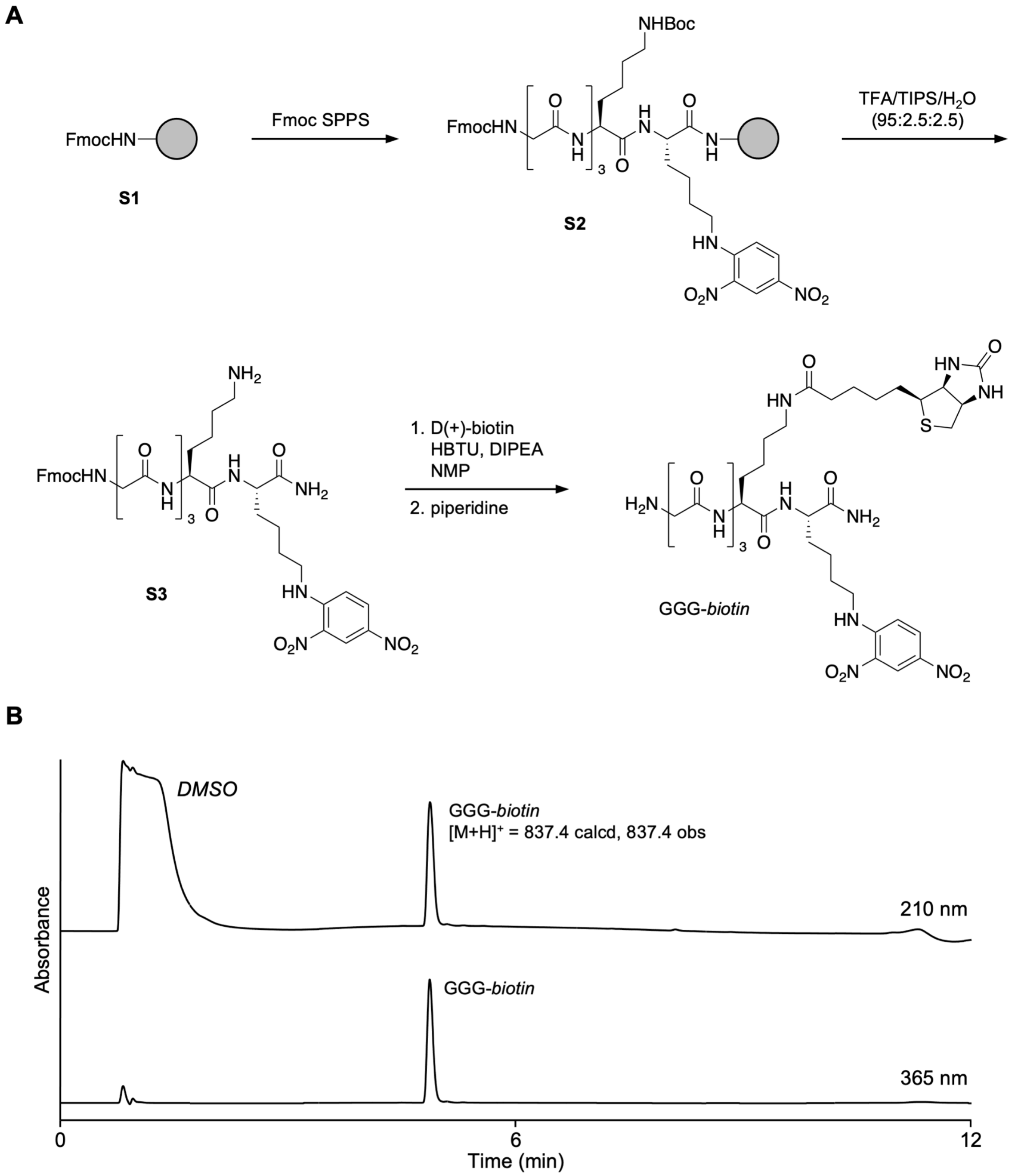

Figure S7. Synthetic scheme and characterization of GGG-*biotin*. (**A**) Synthetic scheme for preparation of GGG-*biotin*. (**B**) RP-HPLC and LC-MS characterization of GGG-*biotin*. The presence of a peak in the 365 nm chromatogram is consistent with the expected absorbance of the 2,4-dinitrophenyl (Dnp) chromophore. Calculated and observed masses represent $[M+H]^+$ ions (monoisotopic).

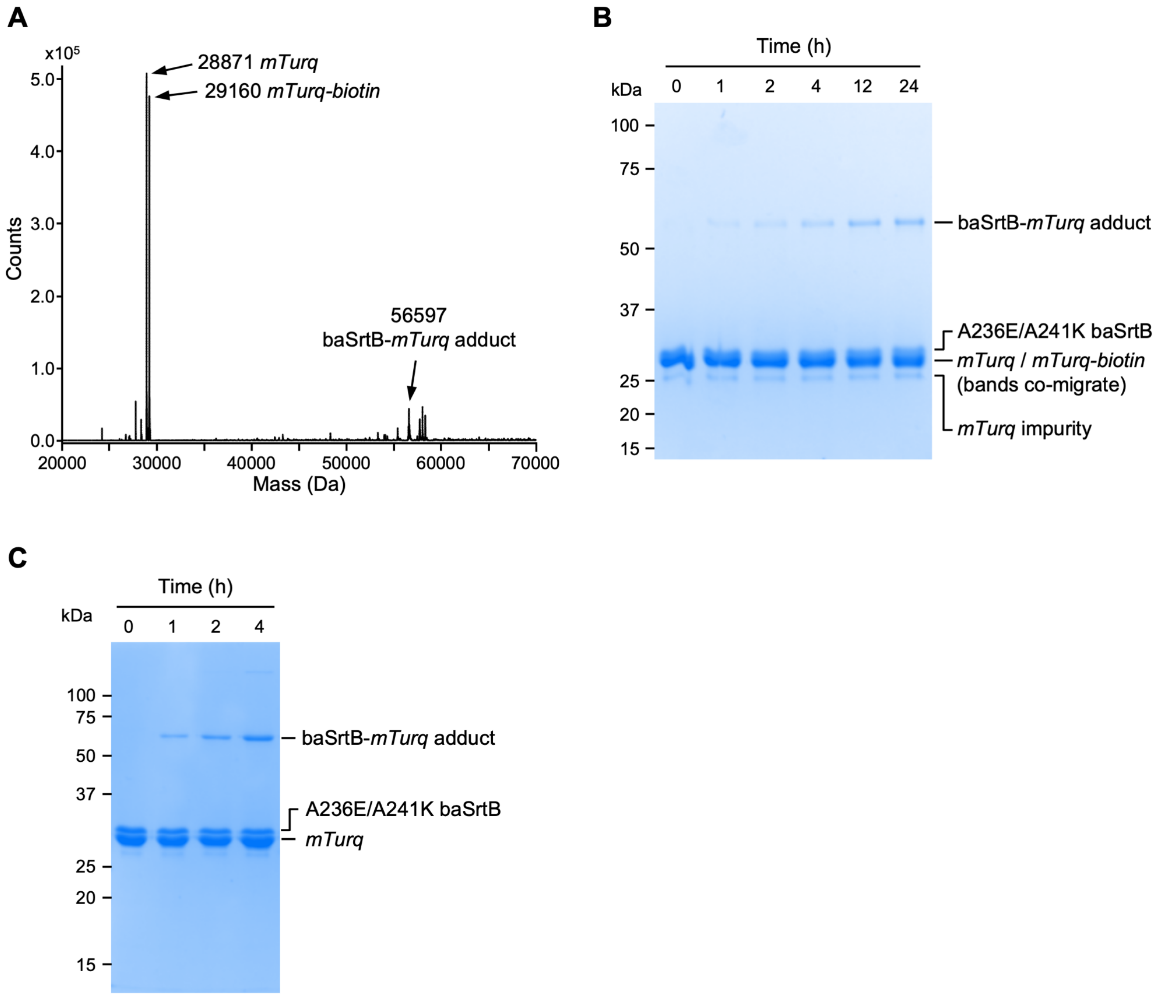

Figure S8. LC-MS and SDS-PAGE characterization of mTurq-DNPKTGDEGGGG ligation to GGG-*biotin*. (**A**) Deconvoluted mass spectrum showing an expanded mass range for the sortase-mediated ligation of 200 μM mTurq-DNPKTGDEGGGG (*mTurq*) and 1 mM GGG-*biotin* in the presence of 50 μM A236E/A241K baSrtB. Data represents the 21 h reaction time point (room temperature). A small signal corresponding to an adduct (baSrtB-*mTurq*) formed following the initial reaction between mTurq-DNPKTGDEGGGG and A236E/A241K baSrtB was observed at 56597 Da. This mass is consistent with the acylation of the baSrtB enzyme by an mTurq-DNPKT fragment (calculated MW for *mTurq* = 28870 Da, calculated MW for *mTurq-biotin* = 29159 Da, calculated MW for baSrtB-*mTurq* adduct = 56597 Da). (**B**) SDS-PAGE analysis of the sortase-mediated ligation reaction described in (**A**). At later time points a higher molecular weight species is observed that is consistent with the formation of a covalent adduct (baSrtB-*mTurq)*. SDS-PAGE was unable to resolve the bands for the mTurq-DNPKTGDEGGGG substrate (*mTurq*) and the biotinylated product (*mTurq-biotin*) formed after sortase ligation. (**C**) Separate SDS-PAGE analysis of control reactions between mTurq-DNPKTGDEGGGG (*mTurq*) and A236E/A241K baSrtB revealed formation of a higher molecular adduct (baSrtA-*mTurq*) in the absence of GGG-*biotin*.

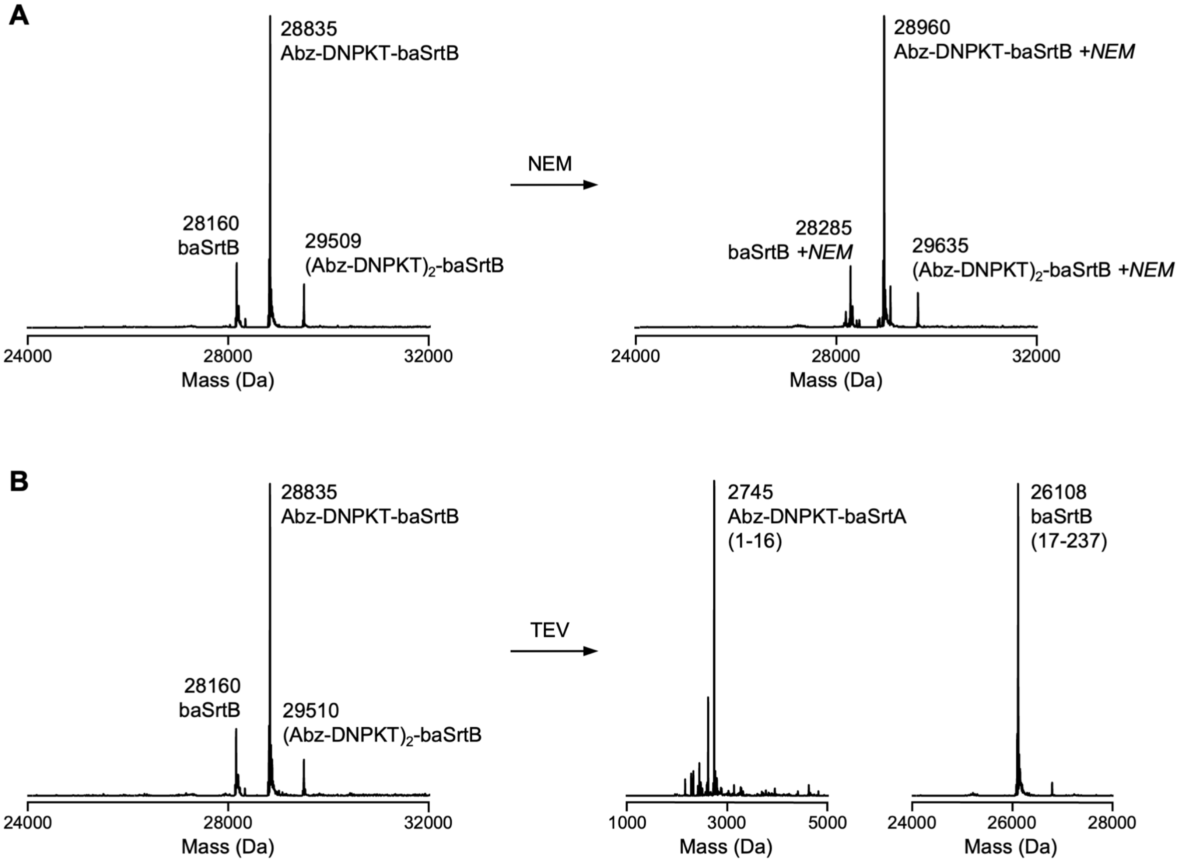

Figure S9. LC-MS characterization of baSrtB enzyme-substrate adducts. (**A**) Deconvoluted mass spectrum (*left*) showing the formation of covalent adducts following the incubation of wild-type baSrtB (50 μM) and Abz-DNPKTGDE-K(Dnp)-*NH₂* (200 μM) for 27 h at room temperature (calculated MW for unmodified baSrtB = 28159 Da, calculated MW for single adduct Abz-DNPKT-baSrtB = 28834 Da, calculated MW for double adduct (Abz-DNPKT)$_2$-baSrtB = 29509 Da). Following incubation with 1 mM NEM for 1 h at room temperature (*right*), all species exhibit a mass increase consistent with NEM alkylation (expected Δ = +125 Da), suggesting that all protein components possess a free catalytic cysteine residue. (**B**) Deconvoluted mass spectrum (*left*) showing the formation of covalent adducts following the incubation of wild-type baSrtB (50 μM) and Abz-DNPKTGDE-K(Dnp)-*NH₂* (200 μM) for 24 h at room temperature. Following incubation with TEV protease for 1 h at room temperature, fragments consistent with modification of the baSrtA N-terminus were formed (*right*). This includes an unmodified fragment consisting of residues 17-237 of wild-type baSrtB (residue number based on the sequence of the recombinant His-tagged construct, calculated MW = 26107 Da). In addition, a modified fragment corresponding to residues 1-16 of baSrtB ligated to Abz-DNPKT (calculated MW = 2745 Da) was observed.

Sequences used for AlphaFold2 modeling. All sequences are in the format: substrate-**$(G_4S)_2$**-SrtB. Accession numbers used for each sequence are in the Materials and Methods. Membrane localization sequences that are subsequently cleaved by a signal peptidase for saIsdC and baIsdC are in italics.

>saIsdC-$(G_4S)_2$-saSrtB
*MKNILKVFNTTILALIIIIATFSNSANA*ADSGTLNYEVYKYNTNDTSIANDYFNKPAKYIKKNGKLYVQITVNHSHW
ITGMSIEGHKENIISKNTAKDERTSEFEVSKLNGKIDGKIDVYIDEKVNGKPFKYDHHYNITYKFNGPTDVAGANAP
GKDDKNSASGSDKGSDGTTTGQSESNSSNKDKVENPQTNAGTPAYIYAIPVASLALLIAITLFV**GGGGSGGGGS**FLT
IVQILLVVIIIIFGYKIVQTYIEDKQERANYEKLQQKFQMLMSKHQEHVRPQFESLEKINKDIVGWIKLSGTSLNYP
VLQGKTNHDYLNLDFEREHRRKGSIFMDFRNELKNLNHNTILYGHHVGDNTMFDVLEDYLKQSFYEKHKIIEFDNKY
GKYQLQVFSAYKTTTKDNYIRTDFENDQDYQQFLDETKRKSVINSDVNVTVKDRIMTLSTCEDAYSETTKRIVVVAK
IIKVS

>baIsdC-$(G_4S)_2$-baSrtB
*MRKISVLPAFIITFVCMLAFLVMPYGKVS*AQLADGTYDINYVIQKAENDSASMANDYFEKPAKLVVKNGEMRVQIPM
NHSAWITEFKAPENGNFVDAKVVNKDESADKRTVEFKIDDLSKPAAAKIHVVVPNVNYDHNYTIRFAFDANVKAVGG
ENKATAVTKNNDQTKTDTKVKEEVKKEESKEVNKEANKGTNESGKAEKTDNPKTGDEARIGLFAALILISGVFLI**GG**
GGSGGGGSSSEKERKKKIFFQRILTVVFLGTFFYSVYELGDIFMDYYENRKVMAEAQNIYEKSPMEEQSQDGEVRKQ
FKALQQINQEIVGWITMDDTQINYPIVQAKDNDYYLFRNYKGEDMRAGSIFMDYRNDVKSQNRNTILYGHRMKDGSM
FGSLKKMLDEEFFMSHRKLYYDTLFEGYDLEVFSVYTTTTDFYYIETDFSSDTEYTSFLEKIQEKSLYKTDTTVTAG
DQIVTLSTCDYALDPEAGRLVVHAKLVKRQ

>Lmo2186-$(G_4S)_2$-lmSrtB
MKKVLVFAAFIVLFSFSFLSTGLTAQAALKDGTYSVDYTVIQGDSDSASMANDYFDKPATVTVNGGKSTVSLQVNHS
KWITGLWVEGNAVSVTSKNASSDTRKVSFPVSTLSNPVNAKIKVDIDDDDLNYHHEYQIKLRFDEGSAKALAGAVKS
SDNNTTTPATKSDSSNKVTNPKSSDSSQMFLYGIIFVATGAGLILL**GGGGSGGGGS**LTLVVLGVFLFSGWKIGMELY
ENKHNQTILDDAKAVYTKDAATTNVNGEVRDELRDLQKLNKDMVGWLTIIDTEIDYPILQSKDNDYYLHHNYKNEKA
RAGSIFKDYRNTNEFLDKNTIIYGHNMKDGSMFADLRKYLDKDFLVAHPTFSYESGLTNYEVEIFAVYETTTDFYYI
ETEFPETTDFEDYLQKVKQQSVYTSNVKVSGKDRIITLSTCDTEKDYEKGRMVIQGKLVTK

>Lmo2185-$(G_4S)_2$-lmSrtB
MKKLWKKGLVAFLALTLIFQLIPGFASAADSRLKDGGEYQVQVNFYKDNTGKTTKESSEADKYIDHTATIKVENGQP
YMYLTITNSTWWQTMAVSKNGTRPEKPAQADVYQDRYEDVQTVSTDAAKDTRVEKFKLSSLDDVIFSYMHIKVDAIS
YDHWYQVDLTIDPSTFKVISEPAVTTPVTLSDGIYTIPFVAKKANDDSNSSMQNYFNNPAWLKVKNGKKMVAMTVND
NKTVTALKTTLAGTLQDVKVVSEDKDANTRIVEFEVEDLNQPLAAHVNYEAPFNGSVYKGQADFRYVFDTAKATAAS
SYPGSDETPPVVNPGETNPPVTKPDPGTTNPPVTTPPTTPSKPAVVDPKNLLNNHTYSIDFDVFKDGTTETSMMESY
VMKPALIKVENNQPYVYLTLTNSSWIKTFQYKVNGVWKDMEVVSGDINKNTRTVKYPVKDGTANTDVKTHVLIEDMP
GFSYDHEYTVQVKLNAATIKDITGKDVTLKEPVKKDILNTGNVASNNNAGPKLAKPDFDDTNSVQKTASKTEKNAKT
NDSSSMVWYITLFGASFLYLAYRL**GGGGSGGGGS**LTLVVLGVFLFSGWKIGMELYENKHNQTILDDAKAVYTKDAAT
TNVNGEVRDELRDLQKLNKDMVGWLTIIDTEIDYPILQSKDNDYYLHHNYKNEKARAGSIFKDYRNTNEFLDKNTII
YGHNMKDGSMFADLRKYLDKDFLVAHPTFSYESGLTNYEVEIFAVYETTTDFYYIETEFPETTDFEDYLQKVKQQSV
YTSNVKVSGKDRIITLSTCDTEKDYEKGRMVIQGKLVTK

References

1. Pichlo, C., Juetten, L., Wojtalla, F., Schacherl, M., Diaz, D., and Baumann, U. (2019) Molecular determinants of the mechanism and substrate specificity of *Clostridium difficile* proline-proline endopeptidase-1. *J. Biol. Chem.* **294**, 11525–11535
2. Carmona, A. K., Schwager, S. L., Juliano, M. A., Juliano, L., and Sturrock, E. D. (2006) A continuous fluorescence resonance energy transfer angiotensin I-converting enzyme assay. *Nat. Protoc.* **1**, 1971–1976
